# Reply to the Letter: “Non-Syndromic elevated IgE”: In reply to: Özdemir Ö. Non-syndromic elevated IgE. World Allergy Organization Journal 2026; 19:101420. Concerning: Castagnoli R, Pecoraro L, Mastrorilli C, et al. Non-syndromic hyper-IgE in children: a practical approach. World Allergy Organization Journal 2026; 19(3):101346

**DOI:** 10.1016/j.waojou.2026.101463

**Published:** 2026-09-11

**Authors:** Riccardo Castagnoli, Luca Pecoraro, Elio Novembre

**Affiliations:** a Pediatric Unit, Department of Clinical, Surgical, Diagnostic and Pediatric Sciences, University of Pavia, Pavia, Italy; b Pediatric Clinic, Fondazione IRCCS Policlinico San Matteo, Pavia, Italy; c Department of Experimental Medicine, Pediatric Section, University of Salento, Hospital “Vito Fazzi”, Lecce, Italy; d Department of Health Sciences, University of Florence, Florence, Italy

Dear Editor,

We thank Professor Özdemir for his careful reading of our review, “Non-syndromic hyper-IgE in children: A practical approach,”^1^ and for the thoughtful comments raised in his Letter to the Editor.^2^ We welcome the opportunity to clarify several points and to expand on aspects that, as he rightly notes, deserve further emphasis. We address each issue in turn.

First, regarding the definition and the term “non-syndromic hyper-IgE.” We agree that terminology in this area is not standardized, and we are grateful for the opportunity to further clarify our operational framework. As noted in the introduction of our review, we deliberately used 2 distinct concepts. “Elevated (total) serum IgE” denotes any value above the age-specific reference interval.^3^ “Hyper-IgE,” by contrast, refers to the more stringent, age-independent threshold of serum IgE exceeding 2000 IU/mL, a cut-off widely used to flag markedly elevated values that warrant a structured differential diagnosis.^4^ The qualifier “non-syndromic” was intended to separate this laboratory finding from the monogenic “hyper-IgE syndromes (HIES),” which we and others have reviewed elsewhere.^5^ We acknowledge that “non-syndromic hyper-IgE” is a descriptive rather than an indexed term and that it does not, at present, retrieve dedicated PubMed entries; our aim was precisely to give practical shape to a differential that is otherwise dispersed across the literature. We hope this clarification resolves the ambiguity.

Second, on the parallel use of “elevated IgE” and “hyper-IgE” in the figures and their captions, Professor Özdemir is correct that both expressions appear in the manuscript, and we appreciate his attention to this. We retained “non-syndromic hyper-IgE” in the caption to align with the article title, whereas within the figure itself we used “elevated IgE,” because we believe that a diagnostic evaluation following our algorithm may be warranted even in children who do not reach the 2000 IU/mL cut-off; this is, indeed, a grey zone. In this context, the title deliberately parallels that of our companion review on inborn errors of immunity with atopic phenotypes,^5^ to underscore the conceptual pairing of “syndromic” (HIES) and “non-syndromic” causes. We regard his proposal of “Non-syndromic elevated IgE” as a reasonable alternative, and we thank him for prompting us to state this rationale explicitly for readers.

Third, regarding the age-specific reference ranges, we welcome the opportunity to clarify the point raised by Professor Özdemir. The values in Table 1 are the age-specific reference intervals established by Martins et al,^3^ who defined them as the central 95% reference interval — that is, the interval bounded by the 2.5th and 97.5th percentiles — derived by non-parametric analysis in accordance with CLSI C28-A guidelines, with transformed parametric analysis applied only to the youngest (6 months–1 year) and the 3-year strata because their sample size was below 120. These intervals therefore represent neither a minimum-to-maximum range nor a symmetric mean ± 2 standard deviations (SD), but the 95% reference interval; because total serum IgE is approximately log-normally distributed, the upper reference limit (the 97.5th percentile for age) corresponds closely to 2 SD above the geometric mean. Accordingly, in our review “elevated IgE” denotes a total serum IgE concentration exceeding this age-specific upper reference limit — a finding conceptually distinct from “hyper-IgE,” the separate, age-independent threshold of >2000 IU/mL. We are glad to make this methodological point explicit.

Fourth, on the absence of frequency data for the individual causes, this is a valuable point. Our objective was to provide a mechanism-oriented differential and a stepwise algorithm rather than an epidemiological synthesis, in part because robust and comparable frequency estimates in children are scarce and strongly context-dependent. As we emphasized, the relative weight of the causes varies markedly with geography: parasitic infection is the leading cause of markedly elevated IgE in resource-limited settings, whereas allergic disease predominates in resource-abundant ones. The single-centre pediatric series cited by Professor Özdemir,^6^ in which atopic disease accounted for approximately 77% of cases, HIES for about 8%, malignancy for 3%, and parasitic disease for only 1.5%, illustrates both the predominance of atopy in that setting and the difficulty of generalizing such frequencies across populations with different exposures. More recently, a dedicated study has specifically examined the aetiology of elevated total serum IgE in children, complementing these observations.^7^

Fifth, regarding bacterial infections, we would note that mycobacterial infections — both *Mycobacterium tuberculosis* and *Mycobacterium leprae* — are addressed in our review and are, of course, bacterial; the associated elevation of IgE is therefore discussed. We agree, however, that pyogenic and other non-mycobacterial bacterial associations received limited attention, and we thank Professor Özdemir for highlighting this. The relationship between ordinary bacterial infection and elevated IgE is less consistent and less well characterized than that observed with helminthic infection, which is why it was not emphasized; nonetheless, selected contexts are instructive. Beyond mycobacteria, a robust and clinically relevant bacterial contribution to IgE responses is exemplified by *Staphylococcus aureus*: specific IgE directed against staphylococcal enterotoxins, which act as superantigens, has been identified as an independent risk factor for asthma and is associated with disease severity in asthma, chronic rhinosinusitis with nasal polyps, and atopic dermatitis.^8^ The case he cites — combined MYD88 and CARD9 deficiency presenting with pyogenic bacterial infection, elevated IgE, and persistent EBV viremia^9^ — is an apt example, although it also illustrates how susceptibility to bacterial infection and hyper-IgE may co-segregate within an underlying inborn error of immunity. This reinforces the central message of our review: markedly elevated IgE accompanied by recurrent or unusual infections should prompt consideration of IEI.

Sixth, on the relationship between IgE and eosinophilia and its diagnostic value, this is an important observation. Eosinophilia frequently accompanies elevated IgE across atopic, parasitic, neoplastic, and immunodysregulatory conditions, and the pattern — in particular the magnitude of the eosinophilia and its persistence — carries diagnostic weight; indeed, marked eosinophilia (>1500 cells/mm^3^) is among the red flags summarized in Table 3 of our review. We would caution, however, that the co-occurrence of elevated IgE and eosinophilia above any given threshold is suggestive but not specific for atopy: the same combination is seen in helminthic infection, in several inborn errors of immunity with immune dysregulation, and in certain malignancies. It is precisely this lack of specificity that motivates the stepwise, red-flag-guided approach we propose, in which the clinical history, physical examination, and broader laboratory context — rather than any single biomarker or threshold — direct the evaluation. This principle is echoed in a recent pediatric algorithm, which likewise concludes that IgE and eosinophil cut-offs, although useful for flagging monogenic disorders, may be misleading in severe atopic dermatitis.^10^

In closing, we are grateful to Professor Özdemir for his generous appraisal of our work and for comments that have provided an opportunity to further clarify our terminology and the rationale underlying our diagnostic approach. Our review was intentionally conceived as a practical, clinically oriented resource for a broad pediatric readership, including general pediatricians who are often the first physicians to evaluate children with elevated serum IgE before referral to pediatric allergy or immunology specialists. Accordingly, we deliberately prioritized a clear, structured, and clinically applicable diagnostic framework over an exhaustive discussion of every highly specialized aspect. We hope this exchange will further enhance the practical value of the review and contribute to a clearer, more consistent approach to the evaluation of children presenting with non-syndromic elevation of serum IgE.

Yours sincerely,

Riccardo Castagnoli, Luca Pecoraro and Elio Novembre on behalf of all co-authors,

Rare Allergic Diseases Task Force of the Italian Society of Pediatric Allergy and Immunology (SIAIP).

## Funding

None.

## Author contributions

All named authors contributed to the conception and drafting of this reply, critically revised it for important intellectual content, and approved the final version.

## Disclosure regarding the use of generative AI and AI-assisted technologies

During the preparation of this correspondence, a generative artificial intelligence large-language model was used to assist with drafting and language editing. The authors reviewed and edited the content and take full responsibility for the accuracy and integrity of the final text.

## Declaration of competing interest

The authors declare that they have no relevant conflicts of interest to disclose in relation to this article.
